# Characterization of epitaxial GaAs MOS capacitors using atomic layer-deposited TiO_2_/Al_2_O_3 _gate stack: study of Ge auto-doping and p-type Zn doping

**DOI:** 10.1186/1556-276X-7-99

**Published:** 2012-02-02

**Authors:** Goutam Kumar Dalapati, Terence Kin Shun Wong, Yang Li, Ching Kean Chia, Anindita Das, Chandreswar Mahata, Han Gao, Sanatan Chattopadhyay, Manippady Krishna Kumar, Hwee Leng Seng, Chinmay Kumar Maiti, Dong Zhi Chi

**Affiliations:** 1 Institute of Materials Research and Engineering, A*STAR, (Agency for Science, Technology and Research), 3 Research Link, Singapore 117602, Singapore; 2 School of Electrical and Electronic Engineering, Nanyang Technological University, Nanyang Avenue, Singapore 639798, Singapore; 3 Department of Electronic Science, University of Calcutta, 92-A. P. C. Road, Kolkata 700 009, India; 4 Centre for Research in Nanoscience and Nanotechnology, (CRNN), University of Calcutta, JD-2 Sector III, Kolkata 700 098, India; 5 Department of Electronics and ECE, Indian Institute of Technology, Kharagpur 721302, India

**Keywords:** epitaxial-GaAs, Ge out-diffusion and auto-doping, ALD, high-*k *dielectrics.

## Abstract

Electrical and physical properties of a metal-oxide-semiconductor [MOS] structure using atomic layer-deposited high-*k *dielectrics (TiO_2_/Al_2_O_3_) and epitaxial GaAs [epi-GaAs] grown on Ge(100) substrates have been investigated. The epi-GaAs, either undoped or Zn-doped, was grown using metal-organic chemical vapor deposition method at 620°C to 650°C. The diffusion of Ge atoms into epi-GaAs resulted in auto-doping, and therefore, an n-MOS behavior was observed for undoped and Zn-doped epi-GaAs with the doping concentration up to approximately 10^17 ^cm^-3^. This is attributed to the diffusion of a significant amount of Ge atoms from the Ge substrate as confirmed by the simulation using SILVACO software and also from the secondary ion mass spectrometry analyses. The Zn-doped epi-GaAs with a doping concentration of approximately 10^18 ^cm^-3 ^converts the epi-GaAs layer into p-type since the Zn doping is relatively higher than the out-diffused Ge concentration. The capacitance-voltage characteristics show similar frequency dispersion and leakage current for n-type and p-type epi-GaAs layers with very low hysteresis voltage (approximately 10 mV).

**PACS: **81.15.Gh.

## Introduction

In recent years, there had been increasing interest in the introduction of III-V semiconductors as high-mobility channel materials in nanoscale silicon-based [Si-based] complementary-metal-oxide-semiconductor [CMOS] devices [1-7]. This migration from the present strained Si channels is due to two reasons: First, with the replacement of silicon oxide [SiO_2_] and silicon oxynitride (SiO_x_N_y_) by high-permittivity [high-*k*] dielectrics as the gate insulator [1-8], the choice of a channel material is no longer restricted to Si. Second, the incorporation of appropriate stressors, such as silicon nitride, can enhance both electron and hole mobilities in sub-90-nm devices; there could be scaling limits to such approaches. An inversion n-channel GaAs field effect transistor [FET] with a metal gate high-*k *dielectric was fabricated on GaAs wafers by de Souza et al. [4]. Ye et al. [5] characterized the Al_2_O_3_/GaAs metal-oxide-semiconductor field effect transistor [MOSFET] and found a very high drain current and a relatively high transconductance. Also, the studies on the effect of the atomic layer-deposited [ALD] Al_2_O_3 _blocking layer indicates that it can suppress the growth of an interfacial layer and that the ALD Al_2_O_3 _could reduce the formation of native arsenic oxides to below the detection level of X-ray photoelectron spectroscopy [6].

For high-volume manufacturing, it is of great interest to develop epitaxial III-V high-mobility channel materials on a silicon platform to realize CMOS devices with increased carrier mobility and device flexibility [9-11]. Convergence of the Si and compound semiconductor industries promises the best of both worlds for device manufacturers due to the high performance, flexibility, and enhanced functionality of III-V compounds coupled with the low manufacturing cost and sheer scale of the Si process. In particular, GaAs has received much attention due to its lower effective mass and, hence, an intrinsic superior transport property than Si. Moreover, it is possible to grow epitaxial GaAs [epi-GaAs] on a Si-based CMOS technology-compatible Ge substrate since the lattice parameter of GaAs (0.5653 nm) is almost identical to that of Ge (0.5658 nm), and both have similar thermal conductivity [9]. In addition, Ge has the added advantage of having a high hole mobility of 1,900 cm^2 ^V^-1 ^s^1 ^at 300 K which is about four times higher than that of Si [12]. This suggests the possibility of a heterogeneous integration of GaAs n-channel FETs with Ge p-channel FETs on a common Si platform.

One of the key considerations in fabricating a surface channel MOSFET using epi-GaAs is to achieve a good interface quality between the epi-GaAs substrate and gate oxide, which is vital for the device performance [13]. Fortunately, atomic layer deposition provides a unique opportunity to integrate high-quality gate dielectrics on bulk and epi-GaAs [2,14,15]. It was observed that ALD Al_2_O_3 _provides a better interface with GaAs interface compared with other ALD high-*k *dielectrics [6,8]. Although, by continuing effort on surface passivation, it is possible to grow a high-quality interface with low defect density, the hysteresis voltage for ALD high-*k*/GaAs gate stack is still high [2,7,15]. There are some attempts to achieve low hysteresis voltage using ALD SiO_2_, directly deposited titanium oxide [TiO_2_], and Si passivation on GaAs substrates [16-19]. On the other hand, GaAs grown at its optimum temperature on Ge will result in high Ge contamination, such as auto-doping and formation of Ge-based complexes, as significant Ge atoms will diffuse into the GaAs epilayer during growth. Chia et al. [20] suggested that a thin 10-nm AlAs interfacial layer is sufficient to effectively block the out-diffusion of Ge atoms at a high growth temperature of 650°C, eliminating Ge-based complexes and auto-doping effects in the GaAs layer. It is highly desirable to grow p-type epi-GaAs with good structural and electronic qualities for n-MOSFET device applications. However, to the best of our knowledge, there is no report of a metal-oxide-semiconductor [MOS] capacitor using p-type epi-GaAs grown on Ge substrates.

TiO_2 _[17,18] gate dielectric provides low hysteresis voltage, and thin ALD Al_2_O_3 _is a promising gate dielectric for surface passivation [6,7] as well as improved interface quality. In this paper, we demonstrate ALD TiO_2_/Al_2_O_3 _gate stack on undoped (which is n-type) and Zn-doped (p-type) epi-GaAs grown by metallorganic chemical vapor deposition [MOCVD] technique. The epi-GaAs device characteristics are compared with that of undoped and Zn-doped epi-GaAs for different concentrations. Further, we have identified the minimum Zn dopant concentration required for p-type epi-GaAs substrates. Electrical and physical analyses and simulation using SILVACO software (SILVACO, Inc., Santa Clara, CA, USA) have also been performed to understand the impact of the material and processing conditions for a high-quality gate stack on epi-GaAs substrates and the impact of Ge diffusion on the performance of MOS characteristics. The surface topography of epi-GaAs and high-*k*/epi-GaAs surfaces was examined via atomic force microscopy [AFM]. Interfacial reaction of high-*k*/epi-GaAs and Ge out-diffusion was studied by time of flight secondary ion mass spectrometry [ToF-SIMS] for all the structures. Capacitance-voltage [*C-V*] and current-voltage [*I-V*] characteristics were measured using an Agilent 4284A LCR (Agilent Technologies Inc., Santa Clara, CA, USA) and a Hewlett-Packard 4140B semiconductor parameter analyzer (Hewlett-Packard Company, Palto Alto, CA, USA), respectively

## Experiment

MOS capacitors were fabricated on epi-GaAs substrates. The epi-GaAs substrates were grown at 620°C to 650°C by MOCVD technique. Vicinal Ge (100) substrates with 6° offcut toward the (111) plane were used to ensure that the epitaxial GaAs grown on Ge is free from APD defects. Prior to the growth of GaAs layers, the Ge substrate was heated up to and kept at 650°C for 5 min under H_2 _environment to remove the native oxide layer. Tertiarybutylarsine and trimethylgallium were introduced into the reactor for the growth of the Zn-doped 300-nm-thick GaAs layer at 620°C. For undoped epi-GaAs, the GaAs substrate was grown on Ge(100) samples with an AlAs interfacial layer at 650°C by MOCVD technique. The details of the film growth and their properties are reported elsewhere [20]. The as-grown wafers were then degreased using isopropanol, cleaned in HF solution (1%) for 3 min to remove the native oxide, and then dipped in NH_4_OH solution for 10 min. A thin layer of Al_2_O_3 _was deposited on epi-GaAs using trimethylaluminium (SAFC Hitech, Haverhill, MA, USA; 99.9%) and H_2_O as the precursors in a viscous flow-type (0.6 Torr working pressure) atomic layer deposition equipment (f·XALD ALD equipment, Azimuth Technologies Pte Ltd., Singapore) with a N_2 _flow rate of 50 sccm at 170°C. After that, TiO_2 _films were deposited under similar conditions. Vapors of TiCl_4 _(Merck & Co., Inc., Whitehouse Station, NJ, USA; 99%) and H_2_O precursors were sequentially introduced into the chamber with an exposure time of 0.1 s and purged by 50-sccm N_2 _flow for 10 s between the two exposures. Post-deposition annealing was carried out in a N_2 _ambient at 500°C for 1 min by rapid thermal annealing technique. The Au metal, deposited by sputtering, was used as the gate electrode (area, 7.8 × 10^-3 ^cm^2^). Finally, a low-resistance ohmic back contact was formed by depositing Ti/Pt/Au alloy on the p-GaAs substrate, AuGeNi alloy on the n-GaAs substrate, and Au on the Ge substrate.

## Results and discussion

**Figure 1:**
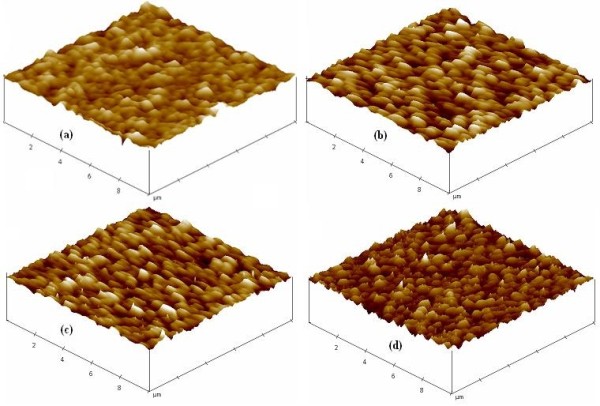
**AFM images (10 × 10 μm^2^) for epitaxial GaAs on Ge structures**. (**a**) Undoped epi-GaAs and Zn-doped epi-GaAs with doping densities of (**b**) 1 × 10^17 ^cm^-3^, (**c**) 1 × 10^18 ^cm^-3^, and (**d**) 1 × 10^19 ^cm^-3^. The rms surface roughness of the structures determined from AFM is (a) 4.0 nm, (b) 4.7 nm, (c) 4.4 nm, and (d) 4.6 nm.

The surface roughness of epi-GaAs and bulk p-GaAs was measured using AFM. Figure 1 shows the surface topology of epi-GaAs on a scale bar of 10 × 10 μm^2^, and a distinct triangular feature is observed. The root-mean-square [rms] surface roughness is high, and it is measured to be 4.0 nm over this scale. These triangular features are typical of epi-GaAs grown on Ge(100) substrates with 6° offcut toward the [111] direction [21]. The rms values for Zn-doped epi-GaAs ranges from 4.4 to 4.7 nm. The ALD high-*k *dielectric stack follows the epi-GaAs topography as observed in the AFM image. The rms value increased slightly to 4.2 nm with ALD coated undoped epi-GaAs after rapid thermal annealing at 500°C in a N_2 _ambient.

**Figure 2:**
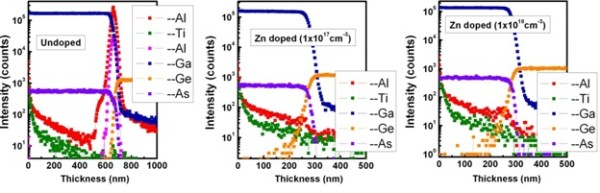
**ToF-SIMS profiles of Ga, As, Al, and Ge atom concentration**. These are the profiles for the epitaxial GaAs layer grown at high temperature on a Ge substrate with and without the AlAs diffusion barrier. The figure shows that the AlAs barrier layer can restrict the Ge diffusion for GaAs/Ge epitaxy.

Figure 2 presents the ToF-SIMS profile of the epitaxial GaAs/Ge interface for undoped and Zn-doped epi-GaAs with ALD coated TiO_2_/Al_2_O_3 _gate stack. The Zn-doped epi-GaAs (without AlAs) was grown at a relatively low temperature (620°C) to suppress Ge out-diffusion. The epi-GaAs thickness is 300 nm. The Ge atoms were diffused up to approximately 100 nm from the GaAs/Ge interface. However, for epi-GaAs films grown at 650°C, Ge is diffused up to 270 nm from the GaAs/Ge interface [20]. It is apparent from the SIMS depth profiles of Ga, As, Ti, Al, and Ge atoms for Zn-doped and undoped epi-GaAs that except for Ge, all other atoms hardly interdiffuse at the heterointerface between GaAs and the Ge substrate. This indicates that Ge is diffused into the epi-GaAs layer, resulting in a strong asymmetry between the two sides of the interface. It is worthy to note that from the SIMS depth profile images, it appeared that diffusion of the Ge atom is of random nature and is independent of Zn concentration. Ge diffusion into the GaAs film is much more pronounced than As/Ga diffusion into the Ge substrate without any diffusion barrier. However, after introducing the AlAs barrier layer, Ge diffusion was mainly confined to the barrier layer. After AlAs interfacial layer insertion, it shows abrupt heterointerfaces, and no significant compositional diffusion of Al and Ge atoms into the GaAs epilayer was observed at a high growth temperature. Since Zn dopant concentration is beyond the SIMS detection limit, it is difficult to see the Zn profile for epi-GaAs.

**Figure 3:**
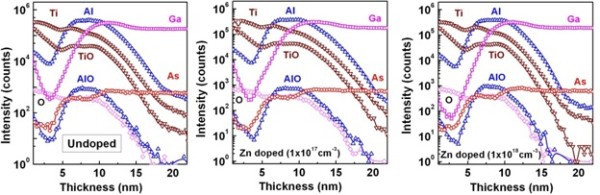
**ToF-SIMS depth profile of elements Ti, Al, AlO, TiO, O, Ga, and As**. These are the depth profile of the elements of the ALD TiO_2_/Al_2_O_3 _dielectric stack on doped (10^17 ^to 10^18 ^cm^-3^) and undoped epi-GaAs structures.

In order to examine the elemental distribution with superior sensitivity and depth resolution, ToF-SIMS measurements on the ALD TiO_2_/Al_2_O_3 _on epi-GaAs substrates were performed. Figure 3 shows the SIMS depth profile from the surface of the ALD TiO_2_/Al_2_O_3 _to the epi-GaAs layer. The transition region of the TiO_2_/Al_2_O_3_/epi-GaAs interface is clearly shown in the Figure 3. A gallium-rich region was observed above the interface. From the gradient of Ti and Al intensity at the TiO_2_/Al_2_O_3 _interface, it is evident that interdiffusion of Ti into Al_2_O_3 _is higher compared to that of Al into TiO_2_. These profiles can be divided into three different sections: In the first region, a decreasing intensity of Ti-, TiO-, and O-related signals is observed. The second region in the ToF-SIMS profile presents Ti and TiO double bumps at the TiO_2_/Al_2_O_3 _interface which exhibit different distributions of Ti inside the layer, i.e., that the stoichiometry is strongly changing with depth. It is important to note that the Al or AlO signal intensity does not vary with depth. This suggests almost constant stoichiometry throughout the Al_2_O_3 _interlayer thickness and aluminum enrichment of the interface. The As and Ga intensity demonstrated a decreasing intensity from the epi-GaAs/high-*k *interface, and no further increase in intensity was observed corresponding to the reduced interfacial As-O or Ga-O layer. The above observation may be explained using the intermixing model proposed by Kamata and Kita et al. [22,23], whereby it is possible that TiO_2_/Al_2_O_3 _was able to mix with residual GaO_x _or out-diffused elemental As such that the TiO_2_/Al_2_O_3_/epi-GaAs stack has an interfacial layer like Ti/Al-GaAsO_x _after annealing.

**Figure 4:**
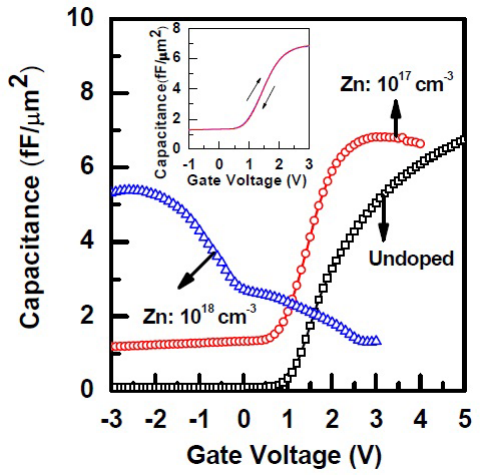
**Capacitance-voltage and hysteresis characteristics of the TiO_2_/Al_2_O_3_/epi-GaAs structure**.

High-frequency *C*-*V *measurements were carried out to evaluate the epi-GaAs substrate doping and electrical properties of the TiO_2_/Al_2_O_3_/epi-GaAs structure. It is known that Ge is an n-type dopant and Zn is a p-type dopant in GaAs. Figure 4 shows the *C*-*V *characteristics of TiO_2_/Al_2_O_3_/epi-GaAs for undoped and Zn-doped epi-GaAs. The *C*-*V *characteristics of epi-GaAs without doping (undoped) and Zn-doped epi-GaAs with a doping concentration of 10^17 ^cm^-3 ^exhibit an n-type behavior since accumulation is achieved at positive gate biases. This is due to the auto-doping of out-diffused Ge atoms in GaAs. On the other hand, epi-GaAs with a Zn dopant concentration of 10^18 ^cm^-3 ^shows a p-type *C*-*V *behavior, which suggests that the concentration of Zn dopant is higher than that of the out-diffused Ge atoms. According to Figure 4, the accumulation capacitance is different for n-type and p-type epi-GaAs substrates. This suggests that the interfacial layer thickness between the high-*k *and semiconductor depends on the nature of the substrate dopants [2]. It is also reported that, in the case of HfO_2 _on Ge substrates, the interface growth kinetics depends on the dopant type [24]. Although, the epi-GaAs MOS capacitor for the Zn-doped epi-GaAs with 10^18 ^cm^-3 ^shows a p-type *C*-*V *behavior, the *C*-*V *curves stretched along the voltage axis. This is due to the presence of defects at the interface. It is not surprising since the structural defects formed near the substrate surface due to the impurity diffusion, particularly when the impurity concentration is high (approximately 1 × 10^18 ^cm^-3^).

It was observed from the SIMS analysis of the epi-GaAs layer grown at 620°C that a significant amount of Ge was diffused into the epi-GaAs thereby converting it to n-type due to auto-doping of Ge to GaAs. By introducing a high density of Zn, it is possible to convert the n-type epi-GaAs to p-type. It is worth noting that although, from SIMS analysis, there was Ge atom diffusion in the epi-GaAs layer up to 20 nm with an AlAs interlayer and up to 100 nm without the AlAs interlayer, however, for both the cases, the epi-GaAs layer shows an n-type behavior. This suggests that although AlAs effectively reduced the Ge atom diffusion into GaAs, there is still some Ge which could possibly be below the SIMS detection limit. From the simulation of Ge diffusion and *C*-*V *characteristics, it was also observed that Ge atoms were present in the epi-GaAs even for the AlAs barrier layer, but the concentration is very low (approximately 10^15 ^cm^-3^). The hysteresis voltage for ALD TiO_2_/Al_2_O_3 _gate stack was very low (approximately 10 mV) as shown in the inset of Figure 4.

**Figure 5:**
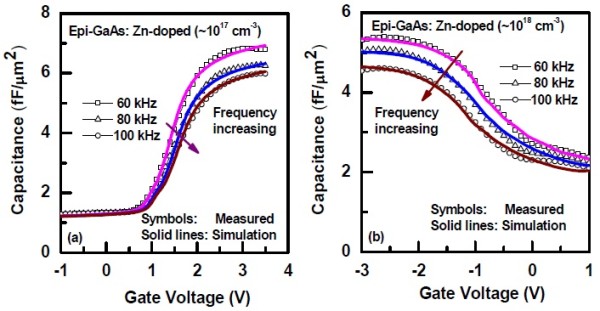
**Frequency dispersion characteristics of the TiO_2_/Al_2_O_3_/epi-GaAs structure**. (**a**) Zn-doped epi-GaAs with a doping concentration of 10^17 ^cm^-3 ^and (**b**) Zn-doped epi-GaAs with a doping concentration of 10^18 ^cm^-3^.

Figure 5 shows the plots of *C*-*V *characteristics of the ALD TiO_2_/Al_2_O_3 _gate stack on Zn-doped epi-GaAs with two different doping concentrations of 10^17 ^cm^-3 ^(Figure 5a) and 10^18 ^cm^-3 ^(Figure 5b). The measured *C*-*V *characteristics were also simulated for all the frequencies considered for a similar structure using SILVACO, a commercially available software package. It is observed that the simulated curves match well with the experimental data. A significant amount of dispersion in the accumulation region of the *C*-*V *curves in the frequency range of 60 to 100 kHz is observed which is attributed to the presence of an interfacial layer with lossy dielectrics. The frequency dispersion for the Zn-doped epi-GaAs with doping concentrations of 10^18 ^cm^-3 ^and 10^17 ^cm^-3 ^is almost similar, and the values are measured to be Δ*C*_60-100 k _which are approximately 15% and 12%, respectively. It is apparent from the ToF-SIMS elemental depth profiles (Figure 3) where a significant amount of interdiffusion of TiO, AlO, O, Ga, and As was noticed. As a result of such interdiffusion, a lossy dielectric layer at the interface has been formed. To account for this, an interfacial layer thickness of 2.6 to 3.0 nm with a dielectric constant of 6 was incorporated during simulation in between the Al_2_O_3_/GaAs layer. The fixed oxide charge densities (*Q*_f_) of 4 × 10^13 ^and -1 × 10^13 ^cm^-2 ^needed to be incorporated to match the flat band voltage. It should be noted that the flat band voltage is negative for the Zn-doped devices with a doping concentration of 10^18 ^cm^-3^, whereas it is positive for 10^17 ^cm^-3 ^doping.

**Figure 6:**
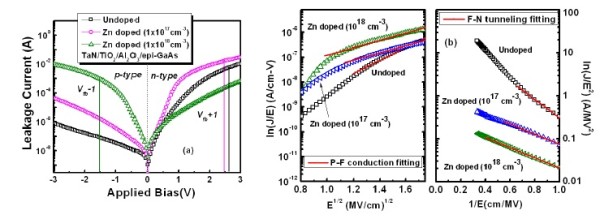
**Gate leakage current and P-F conduction fitting and F-N tunneling fitting**. (**a**) Gate leakage current of the TiO_2_/Al_2_O_3_/epi-GaAs structure for doped and undoped samples. (**b**) P-F conduction fitting and F-N tunneling fitting of *I*-*V *characteristics for different samples.

The leakage current vs. applied voltage [*I*-*V*] characteristics of different MOS capacitors exhibits a leakage current of approximately 10^-3 ^A at flat band voltage, *V*_fb _± 1 V, as shown in Figure 6. It was observed that the leakage current increases sharply with applied voltage and then nearly saturates. Poole-Frenkel [PF] emission was first considered to be the possible conduction mechanism for the leakage current across the gate stack [25]. To clarify whether the leakage current is due to the PF emission, the logarithm of the current density over the electric field was plotted against the square root of electric field as shown in Figure 6b. If the leakage current is governed by the PF emission, such a plot will show a straight line, and from the slope of the straight line, the extracted values of the dynamic dielectric constant are found to be very low compared to the reported results [26,27]. Therefore, we assumed the current conduction in the small electric field is not a pure PF emission, indicating that different conduction mechanisms contribute to the leakage current. Fowler-Nordheim [F-N] tunneling due to the narrowed oxide energy barrier width is also considered as a possible current transport mechanism in the Al_2_O_3_/epi-GaAs heterostructure because the gate leakage mechanism was well fitted by the F-N tunneling model [25,28]. From the slope of the curve, the tunneling barrier heights were found to be 2.1 eV (undoped epi-GaAs) and 1.18 to 1.2 eV (for doped epi-GaAs). The large bandgap of interfacial Al_2_O_3 _and the high-quality oxide film are responsible for this because the F-N tunneling conduction requires sufficient band offsets and a low density of oxide traps [29].

## Conclusions

In summary, epi-GaAs MOS capacitors were fabricated and characterized using electrical and physical analysis. Atomic layer-deposited TiO_2_/Al_2_O_3 _gate stack is used to fabricate epi-GaAs MOS capacitors on a Ge substrate for III-V CMOS applications. The epi-GaAs MOS capacitor shows an nMOS behavior for undoped and even for Zn-doped epi-GaAs with low concentration due to Ge auto-doping, which is confirmed by the SIMS analysis and simulation. Zn-doped epi-GaAs with a high concentration > 10^18 ^cm^-3 ^converts epi-GaAs into p-type. Interfacial reaction mechanisms between epi-GaAs and ALD TiO_2_/Al_2_O_3 _have been discussed through SIMS analysis and capacitance-voltage characteristics. Although the simulated and experimentally obtained *C*-*V *result showed frequency dispersion due to the presence of the interfacial lossy dielectric layer between Al_2_O_3 _and epi-GaAs, the hysteresis voltage for epi-MOS device is very small for the ALD Al_2_O_3_/TiO_2 _gate stack. Therefore, using suitable surface passivation with ALD TiO_2_/Al_2_O_3 _on epi-GaAs can pave the way for the next generation of Si-based CMOS technology for ultrahigh-speed devices or multifunctional devices on a Si platform.

## Competing interests

The authors declare that they have no competing interests.

## Authors' contributions

GKD was involved with the design and planning of the manuscript as well as with the growth and characterization of the ALD TiO_2_/Al_2_O_3 _gate stack on epi-GaAs. TKSW was involved with the surface characterization and participated in the drafting of the manuscript. AFM and electrical measurements were done by YL. CKC was involved with the MOCVD growth of epi-GaAs. Simulation of *C*-*V *characteristics and Ge diffusion was done by AD and SC. CM and CKM were involved with the electrical characterization of the epi-GaAs MOS capacitors. SIMS measurements and analysis were done by HLS. ALD TiO_2_/Al_2_O_3 _was deposited by HG and MKK. DZC was participated in the characterization of the epi-GaAs MOS capacitors. All authors read and approved the final manuscript.

## References

[B1] Bentley SJ Holland M Li X G Paterson GW Zhou H Ignatova O Macintyre D Thoms S Asenov A Shin B Ahn J McIntyre PC Thayne IG Electron mobility in surface- and buried-channel flatband In_0.53_Ga_0.47_As MOSFETs with ALD Al_2_O_3 _gate dielectric IEEE Elect Dev Lett 2011 32 494

[B2] Dalapati GK Tong Y Loh WY Mun HK Cho BJ Electrical and interfacial characterization of atomic layer deposited high-κ gate dielectrics on GaAs for advanced CMOS devices IEEE Trans Elect Dev 2007 54 1831

[B3] Benedicto M Galiana B Molina-Aldareguia JM Monaghan S Hurley PK Cherkaoui K Vazquez L Tejedor P Fabrication of HfO2 patterns by laser interference nanolithography and selectivity dry etching for III-V CMOS application Nanoscale Res Letts 2011 6 400 10.1186/1556-276X-6-400 PMC3211495 21711946

[B4] De Souza JP Kiewra E Sun Y Callegari A Sadana DK Shahidi G Webb DJ Fompeyrine J Germann R Rossel C Marchion C Inversion mode n-channel GaAs field effect transistor with high-k/metal gate Appl Phys Lett 2008 92 153508 10.1063/1.2912027

[B5] Ye PD Wilk GD Yang B Kwo J Chu SNG Nakahara S Gossmann HJL Mannaerts JP Hong M Ng KK Bude J GaAs metal-oxide-semiconductor field-effect transistor with nanometer-thin dielectric grown by atomic layer deposition Appl Phys Lett 2003 83 180 10.1063/1.1590743

[B6] Shahrjerdi D Garcia-Gutierrez DI Tutuc E Banerjee SK Chemical and physical interface studies of the atomic-layer-deposited Al_2_O_3 _on GaAs substrates Appl Phys Lett 2008 92 223501 10.1063/1.2937404

[B7] Lee HD Feng T Yu L Mastrogiovanni D Wan A Gustafsson T Garfunkel E Reduction of native oxides on GaAs during atomic layer growth of Al_2_O_3_ Appl Phys Lett 2009 94 222108 10.1063/1.3148723

[B8] Shi L Liu Z Characterization upon electrical hysteresis and thermal diffusion of TiAl_3_O_x _dielectric film Nanoscale Res Lett 2011 6 557 10.1186/1556-276X-6-557 22011364 PMC3212093

[B9] Suthram S Sun Y Majhi P Ok I Kim H Harris HR Goel N Parthasarathy S Koehler T Acosta T Nishida T Tseng H-H Tsai W Lee J Jammy R Thompson SE Strain additivity in III-V channels for CMOSFETs beyond 22 nm technology node Dig Tech Pap - Symp VLSI Technol 2008 182

[B10] Hill RJW Moran DAJ Li X Zhou H Macintyre D Thoms S Asenov A Zurcher P Rajagopalan K Abrokwah J Droopad R Passlack M Thayne IG Enhancement-mode GaAs MOSFETs with an In_0.3_Ga_0.7_As channel, a mobility of over 5000 cm^2^/V·s, and transconductance of over 475 μS/μm IEEE Electron Device Lett 2007 28 1080

[B11] Passlack M Zurcher P Rajagopalan K Droopad R Abrokwah J Tutt T Park YB Johnson E Hartin O Zlotnicka A Fejes P Hill RJW Moran DAJ Li X Zhou H Macintyre D Thorns S Asenov A Kalna K Thayne IG High mobility III-V MOSFETs for RF and digital applications Tech Dig - Int Electron Devices Meet 2007 621

[B12] Brammertz G Mols Y Degroote S Leys M Sttenbergen JV Borghs G Cyamax M Selective epitaxial growth of GaAs on Ge by MOCVD J Cryst Growth 2006 297 204 10.1016/j.jcrysgro.2006.09.015

[B13] Dalapati GK Chattopadhyay S Kwa KSK Olsen SH Tsang YL Agaiby R Dobrosz P Bull SJ O'Neill AG Impact of strained-Si thickness and Ge out-diffusion on gate oxide quality for strained-Si surface channel n-MOSFETs IEEE Trans Elect Dev 2006 53 1142

[B14] Xuan Y Wu YQ Lin HC Shen T Ye PD Submicrometer inversion-type enhancement-mode InGaAs MOSFET with atomic-layer-deposited Al_2_O_3 _as gate dielectric IEEE Elect Dev Lett 2007 28 935

[B15] Dalapati GK Kumar MK Chia CK Gao H Wang BZ Wong ASW Kumar A Chiam SY Pan JS Chi DZ Interfacial and electrical characterization of atomic-layer-deposited HfO_2 _gate dielectric on high mobility epitaxial GaAs/Ge channel substrates J Electrochem Soc 2010 157 H825 10.1149/1.3453935

[B16] Dalapati GK Chia CK Mahata C Das T Maiti CK Kumar MK Gao H Chiam SY Tan CC Chua CT Cheng YB Chi DZ Surface passivation of GaAs substrates with SiO_2 _deposited using ALD Electrochem Solid-State Lett 2011 14 G52 10.1149/1.3615963

[B17] Dalapati GK Sridhara A Wong ASW Chia CK Lee SJ Chi DZ Characterization of sputtered TiO_2 _gate dielectric on aluminum oxynitride, passivated p-GaAs J Appl Phys 2008 103 034508 10.1063/1.2840132

[B18] Lee MK Yen CF Huang JJ Electrical characteristics of liquid-phase-deposited TiO_2 _films on GaAs substrate with (NH4)_2_S_x _treatment J Electrochem Soc 2006 153 F77 10.1149/1.2181438

[B19] Ok I Kim H Zhang M Kang CY Rhee SJ Choi C Krishnan SA Lee T Zhu F Thareja G Lee JC Metal gate-HfO_2 _MOS structures on GaAs substrate with and without Si interlayer IEEE Electron Device Lett 2006 27 145

[B20] Chia CK Dong JR Chi DZ Sridhara A Wong ASW Suryana M Dalapati GK Chua SJ Lee SJ Effects of AlAs interfacial layer on material and optical properties of GaAs/Ge (100)epitaxy Appl Phys Lett 2008 92 141905 10.1063/1.2908042

[B21] Hudait MK Krupanidhi SB Self-annihilation of antiphase boundaries in GaAs epilayers on Ge substrates grown by metal-organic vapor-phase epitaxy J Appl Phys 2001 89 5972 10.1063/1.1368870

[B22] Kamata Y High-k/Ge MOSFETs for future nanoelectronics Mater Today 2008 11 30

[B23] Kita K Nomura H Nishimura T Toriumi A Impact of dielectric material selection on electrical characteristics of high-k/Ge devices ECS Trans 2006 3 71

[B24] Bai W Lu N Ritenour AP Lee ML Antoniadis DA Kwong DL The electrical properties of HfO_2 _dielectric on germanium and the substrate doping effect IEEE Trans Electron Devices 2006 53 2551

[B25] Sze SM Physics of Semiconductor Devices 2007 3 New York: Wiley

[B26] Tang H Prasad K Sanjines R Schmid PE Levy F Electrical and optical properties of TiO_2 _anatase thin films J Appl Phys 1994 75 2042 10.1063/1.356306

[B27] Mikhelashvili V Eisenstein G Effects of annealing conditions on optical and electrical characteristics of titanium dioxide films deposited by electron beam evaporation J Appl Phys 2001 89 3256 10.1063/1.1349860

[B28] Seo Y Lee S An I Song C Jeong H Conduction mechanism of leakage current due to the traps in ZrO_2 _thin film Semicond Sci Technol 2009 24 115016 10.1088/0268-1242/24/11/115016

[B29] Zhu WJ Ma TP Tamagawa T Kim J Di Y Current transport in metal/hafnium oxide/silicon structure IEEE Electron Device Lett 2002 23 97 10.1109/55.981318

